# Green Extraction of Antioxidant Compounds from Olive Tree Leaves Based on Natural Deep Eutectic Solvents

**DOI:** 10.3390/antiox12050995

**Published:** 2023-04-25

**Authors:** Aina Mir-Cerdà, Mercè Granados, Javier Saurina, Sonia Sentellas

**Affiliations:** 1 Department of Chemical Engineering and Analytical Chemistry, Universitat de Barcelona, Martí i Franquès 1-11, E08028 Barcelona, Spain; 2 Research Institute in Food Nutrition and Food Safety, Universitat de Barcelona, Av. Prat de la Riba 171, Edifici Recerca (Gaudí), E08921 Santa Coloma de Gramenet, Spain; 3 Serra Húnter Fellow Programme, Generalitat de Catalunya, Via Laietana 2, E08003 Barcelona, Spain

**Keywords:** polyphenols, olive tree leaves, natural deep eutectic solvents, green extraction, HPLC-UV-MS/MS, circular economy

## Abstract

Agri-food industries generate a large amount of waste that offers great revalorization opportunities within the circular economy framework. In recent years, new methodologies for the extraction of compounds with more eco-friendly solvents have been developed, such as the case of natural deep eutectic solvents (NADES). In this study, a methodology for extracting phenolic compounds from olive tree leaves using NADES has been optimized. The conditions established as the optimal rely on a solvent composed of choline chloride and glycerol at a molar ratio of 1:5 with 30% water. The extraction was carried out at 80 °C for 2 h with constant agitation. The extracts obtained have been analyzed by high-performance liquid chromatography coupled to tandem mass spectrometry (HPLC-MS/MS) in MRM mode. The comparison with conventional ethanol/water extraction has shown that NADES, a more environmentally friendly alternative, has improved extraction efficiency. The main polyphenols identified in the NADES extract were Luteolin-7-O-glucoside, Oleuropein, 3-Hydroxytyrosol, Rutin, and Luteolin at the concentrations of 262, 173, 129, 34, and 29 mg kg^−1^ fresh weight, respectively.

## 1. Introduction

Polyphenols, characterized by having more than one phenol group in their structure, are one of the most relevant families of secondary metabolites in plants. These compounds have antioxidant, anti-inflammatory, and antimicrobial properties, and for this reason, they have been linked to positive effects on diseases such as cancer, diabetes, hypertension, and cardiovascular diseases, among others [1,2]. Phenolic compounds are obtained from the diet, mainly from fruits, vegetables, and beverages such as tea, coffee, and wine. Nowadays, nutraceutical tablets rich in one or several polyphenols are also marketed [3]. In addition, within the framework of the circular economy, agri-food industry residues are seen as a potential source of polyphenolic compounds; thus, in the last years, special interest in reusing these wastes has arisen.

Among agri-food residues, those derived from the production of olive oil are of special interest. The olive tree is one of the main crops in the Mediterranean area that concentrates about 60% of the world’s olive production. Spain, Italy, and Greece stand out as olive European-producing countries. Indeed, Spain is currently the world’s leading producer, with 45% of the global production [1,4]. In 2019, more than 1.1 million tons of olive oil were produced in Spain [4] (official data from the Food and Agriculture Organization of the United Nations, FAO), generating lots of olive by-products, including olive oil pomace and olive tree leaves. In general, compounds such as 3-Hydroxytyrosol, Oleuropein, and Oleuropein derivatives, which are abundant and characteristic in olive oils, are also remarkable in related waste matrices, specifically in olive tree leaves [5,6,7]. Consequently, these residues can be regarded as a valuable source of phenolic compounds [8,9].

Solid-liquid extraction with hydro-organic mixtures is the procedure most widely used for the extraction of phenolic compounds from agri-food waste [7,10,11]. However, the solvents used as extractants are mostly restricted to water-ethanol due to toxicological and environmental issues. Apart from these conventional solvents, deep eutectic solvents (DES) are gaining popularity in the polyphenol extraction field [12,13,14,15]. DESs consist of mixtures of two types of components: an H-bond acceptor compound (HBA) and an H-bond donor compound (HBD). The resulting mixture is characterized by a lower melting point than one of the individual components [16]. This behavior was first reported by Abbott et al. when they observed that a 1:2 molar ratio mixture of choline chloride (melting temperature, 302 °C) and urea (melting temperature, 133 °C) was liquid at room temperature [17]. In addition, the resulting solvent has an enhanced extraction power for both polar and non-polar compounds. Within this class of solvents, the subgroup called “natural” deep eutectic solvents (NADES) consists of DES, in which both components are primary metabolites. These mixtures are mainly based on choline derivatives as the H-bond acceptor and saccharides, organic acids, or urea as the H-bond donor [18,19,20]. NADES are biodegradable, non-toxic, easy to prepare, and relatively inexpensive solvents with high thermal stability and low volatility [16,21]. The major drawback of NADES is their high viscosity which may hinder the whole extraction process. To overcome this, water or ethanol is commonly added to the mixture [22].

Currently, when green reagents and procedures are increasingly demanded, the recovery of bioactive compounds from waste matrices, the scientific community is ever more involved in reaching a suitable compromise between the green and ecological nature of production processes and their chemical efficiency [23,24]. In this sense, the use of different NADES for the extraction of polyphenols from a wide variety of plant products seems to be a good alternative [12,15]. Regarding the extraction of polyphenols from olive tree leaves, few studies have been conducted using NADES as an extractant solvent, pointing out the need for additional investigations to evaluate the performance of these solvents in the recovery of polyphenolic compounds from this by-product. Indeed, NADES generated from different components has been evaluated to improve the extraction, in most cases, in terms of global parameters, such as antioxidant capacity or the total content of polyphenols determined by the Folin–Ciocalteu assay. However, it is challenging to conclude which is the best system without knowing how some of the most relevant compounds of the matrix behave. For instance, Boli et al. and de Almeida Pontes et al. used choline chloride:acetic acid NADES to increase the phenolic content (based on the Folin–Ciocalteu index) and the antioxidant capacity with respect to the extraction with ethanol [6,22]. L-Lactic acid combined with glycerol or glycine has also been demonstrated to be suitable for obtaining Oleuropein-rich extracts [25,26]. Contrarily, the extraction performance of Oleuropein and other polyphenols, namely 3-Hydroxytyrosol, Luteolin-7-O-glucoside, and Rutin using glycerol as HBD and lysine as HBA, similarly resulted in conventional extraction while this NADES boosted the extraction of tyrosol [27].

The objective of this work is to explore the possibilities of NADES for the recovery of polyphenols from olive tree leaves generated as waste during the production of extra virgin olive oil. The main polyphenols extracted from olive tree leaves were first elucidated to achieve this objective. Then, different choline-based NADES and extraction conditions were assessed by comparing the antioxidant capacity and the polyphenolic profile of the NADES extracts with a conventional hydro-organic extract. The antioxidant capacity was based on the ferric-reducing antioxidant power (FRAP) and the 2,2-diphenyl-1-picrylhydrazyl-hydrate free radical (DPPH) assays and liquid chromatography (LC) with ultraviolet detection and coupled to mass spectrometry (LC-UV-MS and LC-UV-MS/MS) were used for profiling. From these results, the potential of NADES for the extraction of polyphenols from olive tree leaves has been demonstrated as an alternative, in terms of both improved extraction efficiency and the method’s greenness, to traditional solvents.

## 2. Materials and Methods

### 2.1. Olive Leaf Samples

Olive tree (*Olea europaea* L.) leaves of different varieties (*Arbequina*, *Verdiella*) were collected during the production process of extra virgin olive oil in Albelda (Huesca, Spain) in November 2021. Once the leaves arrived at the laboratory, they were crushed with a grinder until a small particle size was obtained (<2 mm). The obtained powder was stored at −18 °C until being used.

### 2.2. Chemicals and Reagents

Standards of 55 phenolic compounds were used for identification and quantification purposes. Gallic acid, Caffeic acid, Ferulic acid, Vanillic acid, Ethyl gallate, Ellagic acid, Epicatechin, Syringic acid, 4-Hydroxybenzoic acid, p-Coumaric acid, 3-Methylcatechol, 4-Ethylcatechol, 2,5-Dihydroxybenzoic acid, 4-Methylcatechol, 3,4-Dihydroxybenzoic acid, Apigenin, Luteolin-7-O-glucoside, Oleocanthal, Oleacein, and Synapic acid were from Sigma Aldrich (St. Louis, MO, USA); Resveratrol, Catechin, Rutin, Myricetin, 3-Hydroxytyrosol, and Verbascoside were from TCI (Tokyo, Japan); Hesperidin and Hesperetin from Glentham (Wiltshire, UK); Quercetin, Chlorogenic acid, Chrysin, and Fisetin were from Merck (Darmstadt, Germany); Epigallocatechin, Naringenin, Luteolin, Oleuropein, and Oleuropein aglycone from Biosynth Carbosynth (Berkshire, UK); trans-Coutaric acid and Caftaric acid were from Phytolab (Vestenbergsgreuth, Germany); Diosmin, Catechol, Tyrosol were from Alfa Aesar (Kandel, Germany); Naringin and Procyanidin C1 were from TargetMol (Boston, MA, USA); Vanillin, Quercetin-3-O-glucoside, Kaempferol, and trans-cinnamic acid were from Fluka (Buchs, Switzerland); Pinocembrin were from Thermo Fisher Scientific (Waltham, MA, USA); Galangin were from CymitQuímica (Barcelona, Spain); Procyanidin A2, Procyanidin B1, Procyanidin B2, and Procyanidin C2 were from Extrasynthese (Genay, France); and Astilbin was from BioPurify (Chengdu, China).

Choline chloride (ChCl, Thermo Fisher, Kandel, Germany), glycerol (Gly, Thermo Fisher, Kandel, Germany), urea (Thermo Fisher, Kandel, Germany), and lactic acid (Lac, Acros organics, Geel, Belgium) were used to prepare NADES.

Reagents for the spectrophotometric determination of the antioxidant capacity were as follows: FeCl_3_, HCl (37%, *v*/*v*), sodium hydrogen phosphate, and potassium dihydrogen phosphate (Merck, Darmstadt, Germany); formic acid was from Sigma-Aldrich (St. Louis, MO, USA), and Fe (III)-2,2,6-tripyridyl-s-triazine (TPTZ) and 2,2-diphenyl-1-picrylhydrazyl (DPPH) were from Alfa Aesar (Kandel, Germany). Trolox from Carbosynth (Berkshire, UK) was used for calibration purposes. Other solvents used were ethanol (EtOH) and dimethylsulfoxide (DMSO > 99% *v*/*v*) from Merck (Darmstadt, Germany), acetonitrile (Panreac, Barcelona, Spain), and purified Milli-Q water (Millipore Corporation, Bedford, MA, USA).

### 2.3. Instruments

A Dionex Ultra High-Performance Liquid Chromatography (UHPLC) system coupled to a Linear Trap Quadrupole (LTQ) Orbitrap Velos mass spectrometer with a HESI-II electrospray ionization source (Thermo Scientific, San Jose, CA, USA) was used for polyphenol identification. LC-High Resolution Mass Spectrometry (LC-HRMS) data were acquired and processed with Xcalibur 2.2 (Thermo Scientific, San Jose, CA, USA).

An Agilent 1100 Series liquid chromatograph (Agilent, Technologies, Palo Alto, CA, USA), coupled to an Applied Biosystems 4000 QTrap hybrid triple quadrupole/linear ion trap mass spectrometer (AB Sciex, Framingham, MA, USA) was used for quantitation purposes. The LC-UV-MS/MS data were acquired and processed with Analyst 1.6.2. (AB Sciex, Framingham, MA, USA).

The spectrometric determination of the antioxidant capacity was performed in an 8453 UV-Vis Spectrophotometer (Agilent, Santa Clara, CA, USA) using QS quartz cuvettes (10 mm optical path) from Hellma Analytics (Jena, Germany).

Additionally, other laboratory equipment comprises a hot plate stirrer with temperature controller (IKA^®^ RCT basic), a Hettich Rotanta 460 RS centrifuge (Tuttlingen, Germany), a Vibra Mix R Vortex (Ovan, Barcelona, Spain), and an ultrasonic cleaner Branson 5510EMTH (Sigma-Aldrich, St. Louis, MO, USA).

### 2.4. NADES Preparation

In order to make a comparative study, NADES based on choline chloride (ChCl) as the HBA and different HBD, such as glycerol (Gly), urea, and acid lactic (Lac), were prepared in standard conditions, i.e., HBA:HBD molar ratios of 1:2 and water mass percentages of 10%. In addition, HBA:HBD molar ratios of 1:2, 1:5, and 2:1 and water mass percentages of 10%, 20%, and 30% were assayed for the ChCl:Gly system. The HBA and HBD components were mixed in the appropriate ratio and heated, with constant stirring, at 60 °C in a water bath until obtaining a colorless and homogeneous liquid. The corresponding percentage of water was then added. The methodology for the preparation of the NADES was based on previous papers by Espino et al. [28] and Ozturk et al. [29] that were adapted to our case.

### 2.5. Extraction of Phenolic Compounds with NADES

The extraction of phenolic compounds from olive tree leaves was carried out as follows: 0.5 g of sample was mixed with 10 mL of NADES. The extraction took place at 80 °C in a water bath for 2 h with constant stirring. The final conditions were optimized with an experimental design, temperature and time being the studied factors. Levels of temperature and time were 40 °C, 60 °C, and 80 °C and 15 min, 30 min, 60 min, and 120 min, respectively. All the conditions were tested in duplicate; thus, a total of 24 experiments were conducted to optimize extraction conditions. Extracts were also prepared with an EtOH/water mixture (20:80, *v*/*v*) to compare the extraction performance of NADES with that of a conventional solvent. For this purpose, 0.5 g of sample was extracted with 10 mL of ethanol/water (20:80, *v*/*v*) in a water bath at 60 °C for 15 min with constant stirring. In any case, the extracts obtained were centrifuged at 3000 rpm for 10 min. The supernatant was filtered through a 0.45 µm nylon filter from Agilent Technologies (Waldbronn, Germany) and placed in an HPLC vial. The extracts were stored in the freezer (−18 °C) until analysis.

### 2.6. Antioxidant Capacity Assays

The FRAP assay was according to Alcalde et al. [30]. Briefly, the FRAP reagent consisted of 20 mmol L^−1^ FeCl_3_, 10 mmol L^−1^ TPTZ (containing 50 mmol L^−1^ HCl), and 50 mmol L^−1^ formic acid solution mixed in the proportion of 1:2:10 (*v:v:v*). Precisely 600 μL of the FRAP reagent and 20 μL of the filtered NADES extract (or 100 μL of the filtered EtOH/water (20:80, *v*/*v*) extract) were mixed and diluted to 5 mL with Milli-Q water. After 5 min, the absorbance was measured at 595 nm using the reagent blank as the reference. For calibration purposes, instead of the sample extracts, appropriate volumes of a Trolox standard solution (100 mg L^−1^) were added to provide concentrations in the range of 0.2–10 mg L^−1^. Results were expressed as mg of Trolox equivalents per kg.

The DPPH method was performed according to Alcalde and colleagues [30]. The stock reagent solution was prepared by dissolving 0.0078 g of DPPH (2,2-diphenyl-1-picrylhydrazyl) in 100 mL of ethanol. This solution was kept in the dark for 2 h. To develop the reaction, 2 mL of the DPPH reagent solution, 1.6 mL of 50 mM phosphate buffer, the required volume of sample extract −20 μL of NADES or 100 μL of EtOH/water (20:80, *v*/*v*) extracts and ethanol up to 4 mL were mixed in amber glass vials. After keeping the mixture for 45 min in the dark, the absorbance was measured at 517 nm. The reagent blank was used as the reference. Trolox was also used as the standard to build the calibration curve prepared as above but replacing the sample extract with appropriate volumes of Trolox standard solution, providing concentrations from 0 to 15 mg L^−1^. Results were expressed as mg of Trolox equivalents per kg of sample.

### 2.7. LC-UV-MS and LC-UV-MS/MS Methods

The resulting extracts were analyzed by liquid chromatography with ultraviolet detection and coupled to mass spectrometry. LC-UV-MS and LC-UV-MS/MS methods previously established by Mir-Cerdà et al. were adapted to this case [31]. Compounds were separated in a Kinetex C18 column (150 mm × 4.6 mm I.D., 2.6 µm particle size) from Phenomenex (Torrance, CA, USA) using 0.1% (*v:v*) formic acid aqueous solution and acetonitrile (ACN) as the components of the mobile phase. The flow rate was 0.7 mL min^−1^, and the elution gradient was as follows: 0 to 10 min, 3% to 15% ACN; 10 to 20 min, 15% to 45% ACN; 20 to 22 min, 45% to 90% ACN; 22 to 24 min, 90% ACN; (column cleaning); 24.0 to 24.2 min, 90% to 3% ACN; and 24.2 to 30 min, 3% ACN (column stabilization). The injection volume was 10 µL. UV absorbance from 190 to 400 nm was recorded.

The polyphenols identification was carried out with HRMS with the LTQ orbitrap using data-dependent acquisition mode. A full scan (from *m*/*z* 110 to 1000) was recorded in negative mode using a resolution of 30,000 fullwidth at half-maximum (FWHM) at *m*/*z* 200. In addition, a data-dependent product ion scan was activated when the full scan signal was higher than 5.0 × 10^3^ (peak intensity threshold). Stepped normalized collision energies (NCE) of 17.5, 35, and 52.5 were applied, and MS/MS were recorded from *m*/*z* 50. Nitrogen (purity higher than 99.98%) was used as HESI-II sheath gas, ion-sweep gas, and auxiliary gas at flow rates of 50, 2, and 20 arbitrary units, respectively. Capillary and S-Lens RF voltages were set at −5 kV and 50 V, respectively. The source temperature was kept at 350 °C, and the capillary temperature was 375 °C. The HRMS analyzer was tuned and calibrated every 3 days by using the calibration solution supplied by Thermo Fisher Scientific.

Low-resolution mass spectrometry, conducted in a 4000 Qtrap spectrometer, was used for structural confirmation and quantitation purposes. Polyphenols were monitored in negative electrospray ionization (ESI) tandem mass spectrometry (MS/MS) in the multiple reaction monitoring (MRM) modes. The ion spray voltage was set at −4500 V. The source temperature was set at 500 °C. Curtain gas, ion source gas 1, and ion source gas 2 consisted of nitrogen and were set at 10, 50, and 50 arbitrary units, respectively. Ion transition pairs, declustering potential (DP), collision energy (CE), collision exit cell potential (CXP), and entrance potential (EP) were optimized for each analyte and are given in Table S1.

### 2.8. Statistical Analysis

The significance of factors and their potential interactions was evaluated by an ANOVA and Students’ *t*-test using Microsoft Excel. The significance level was 0.05.

## 3. Results and Discussion

### 3.1. Identification of Polyphenols in Olive Tree Leaves Extracts

**Figure 1:**
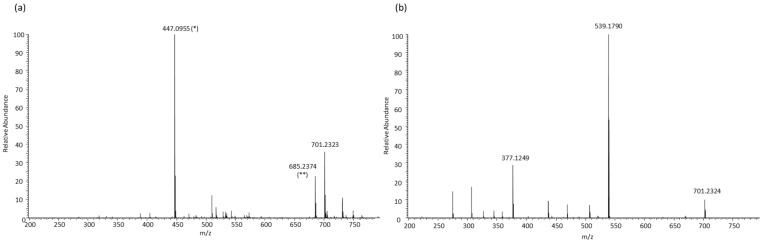
HRMS spectrum of chromatographic peak at 16.69 min (**a**) and HRMS/MS spectrum of ion at *m*/*z* 701.23 (**b**). Peaks corresponding to [M − H]^−^ ion of coeluting compounds were observed in the HRMS spectrum: (*) Luteolin-7-O-glucoside and (**) unknown.

The identification of phenolic compounds was carried out by LC-HRMS/MS using the data-dependent acquisition mode. Table 1 shows the LC-HRMS information and the tentative assignment for all identified compounds. After a tentative annotation based on mass spectra, the identity of the compounds was experimentally confirmed by comparing, when available, retention time, [M − H]^−^ *m*/*z*, and MS/MS data with those of pure standards. As an example, Figure S1 shows the MS and MS/MS spectra of the peak identified as Oleuropein in the sample as well as those for the Oleuropein standard. As can be seen, both [M − H]^−^ and MS/MS fragments in the sample match with those observed for the standard, thus confirming the proposed identification. Those compounds without available standards were tentatively annotated based on the molecular ion and the observed MS/MS fragmentation. This is the case, for instance, of some Oleuropein derivatives. The MS spectrum of the chromatographic peak at 16.69 min shows the ion at *m*/*z* 701.2317, which matches with the [M − H]^−^ ion of Oleuropein glucoside with the mass error of 2.6 ppm (Figure 1a). In addition, the main fragments observed in the MS/MS spectrum at *m*/*z* 539.1790 and 377.1249 may correspond to the loss of one and two glucose moieties, respectively (Figure 1b), which validates the proposed assignment. Similarly, other compounds present in the studied extracts were tentatively identified (Table 1).

### 3.2. Optimization of Extraction Conditions

A preliminary study based on experimental design and using ChCl:Gly (1:2) with 10% water as a model for NADES solvents was carried out to select the extraction technique. Three extraction conditions were compared, namely: (i) ultrasound-assisted extraction (UAE) at a temperature of 60 °C for 1 h, (ii) UAE at 60 °C for 1 h with previous shaking for 10 s with a vortex (UAE + vortex), and (iii) extraction assisted by magnetic stirring at 60 °C for 1 h. All the conditions were assayed in triplicate (*n* = 3), and the extraction efficiency was evaluated by determining the antioxidant activity through the FRAP and DPPH indices. Thus, the antioxidant capacity, from lowest to highest, is as follows: UAE < UAE + vortex < magnetic stirring, suggesting noticeable differences in extraction efficiency. In the case of FRAP, the antioxidant capacity of the extract obtained by magnetic stirring increased by 80% and 110% compared with UAE + vortex and UAE, respectively. For DPPH, antioxidant activity is at least 50% higher when magnetic stirring is used. This behavior is probably due to the high viscosity of NADES that hinders the proper ultrasonic cavitation and decreases the mass transport efficiency. Then, a magnetic stirring is needed to obtain high extraction yields. It should be mentioned, however, that the percentage of water and the working temperature has a remarkable influence on this aspect, as the viscosity decays with increasing these factors, and the mass transfer can be enhanced.

NADES have been extensively used for the recovery of bioactive compounds from agri-food residues [19,22]; however, multiple combinations of HBDs and HBAs have been reported to be appropriate. Indeed, the suitability of a specific solvent composition depends on both the intended compounds to be extracted and the matrix. Hence, different solvent components have to be evaluated for each specific case. Here, in order to optimize the extraction of polyphenolic compounds from olive tree leaves, ChCl was selected as the hydrogen bonding acceptor molecule, while various HBD were evaluated, including Gly, Urea, and Lac. For a preliminary assessment of the donor capacity, common conditions were established for the three systems to be studied (ChCl:Gly, ChCl:Urea, and ChCl:Lac), working at an HBA/HBD molar ratio 1:2 with 10% water, and extraction by constant stirring at 60 °C for 1 h. In addition, extractive capacities were also compared to those using a conventional solvent consisting of EtOH/water (20:80, *v*/*v*). This EtOH/water solvent was chosen based on preliminary studies by Tapia et al., in which its composition was optimized to extract the major phenolic compounds in other related matrices from the olive oil production industry [32]. Those samples were rich in tyrosol derivatives and flavonol glycosides with a moderate polarity that was extracted efficiently using the EtOH/water solvent.

The antioxidant capacity measured by the FRAP method and the radical scavenger capacity by the DPPH assay were determined to evaluate the extraction efficiencies. FRAP indexes are 14,200, 18,000, 6600, and 14,400 mg Trolox equivalents kg^−1^ (considering fresh weight, fw) for the ChCl:Gly, ChCl:Urea, ChCl:Lac, and EtOH/water extracts, respectively. ChCl:Lac extractant shows the lowest extraction efficiency, while for the other systems, similar results are obtained. Regarding the DPPH index, values of 11,900, 16,500, 35,000, and 15,000 mg Trolox equivalents kg^−1^ fw are obtained for the ChCl:Gly, ChCl:Urea, ChCl:Lac, and EtOH/water systems, respectively. Again, clear differences can only be ascertained with the ChCl:Lac NADES; however, in this case, it showed the maximum antioxidant capacity. Conclusive results were not obtained by measuring the antioxidant capacity; thus, in order to select the NADES system showing the best extraction efficiencies, the extraction of individual phenolic compounds is evaluated. Table 2 shows the relative concentration (with respect to the concentration in the ChCl:Gly extract) for the main phenolic compounds identified. As can be seen, the extraction of phenolic compounds strongly depends on the solvent system used as the extractant. Comparing the three NADES studied, ChCl:Gly and ChCl:Urea media showed better performances than ChCl:Lac; overall, the ChCl:Lac system is the less efficient solvent for the extraction of polyphenols, except for 3-Hydroxytyrosol and Vanillin, which extraction in the ChCl:Lac NADES was superior to the other solvents. Focusing on the ChCl:Gly and ChCl:Urea extracts, the highest differences are observed for Caffeic acid, Verbascoside, Hesperidin, Quercetin, and Oleuropein aglycone, with concentrations in the ChCl:Urea extract more than ten-fold lower than in ChCl:Gly. For 3-Hydroxytyrosol, Apigenin, Luteolin, or Oleuropein, the concentration is up to 10-fold higher in the ChCl:Gly extract than in the urea counterpart. On the other hand, some compounds are better extracted with the ChCl:Urea solvents, such as 3,4-Dihydroxybenzoic acid, Diosmin, Rutin, Luteolin-7-O-glucoside, or Naringin. However, in these cases, the observed differences are less relevant. The extraction with ChCl:Urea is up to two-fold higher than that with ChCl:Gly. When comparing NADES with the conventional EtOH/water solvent, the latter provides a poorer extractive performance, with concentrations always lower than those found using NADES. In particular, for all the identified compounds, the recovery in this ethanolic solvent is lower than in the ChCl:Gly mixture. For some compounds, such as Oleuropein, Ferulic acid, or Luteolin-7-O-glucoside, the decrease in the performance is moderate, but for others, the recoveries with the hydro-organic solvent are less than 10% of those achieved with ChCl:Gly.

Overall, ChCl:Gly and ChCl:Urea are the systems showing better performances considering the individual compounds to be extracted. In general, focusing on 3-Hydroxytyrosol, Oleuropein, Luteolin, and Oleuropein and luteolin derivatives as the most renowned and characteristic compounds in olive matrices, these compounds are more efficiently extracted with ChCl:Gly; thus, this NADES composition was selected for further studies.

#### ChCl:Gly System Optimization

The NADES composition was evaluated to obtain the maximum extraction efficiencies. Two variables were studied according to a two-factor and three-level experimental design: the molar ratio between both components (ChCl and glycerol) and the percentage of water to be added. A set of nine experiments with three replicates of the central point was designed. Levels of water percentage and ChCl:Gly ratios were 10%, 20%, and 30% water and 2:1, 1:2, and 1:5 molar ratios, respectively.

**Figure 2:**
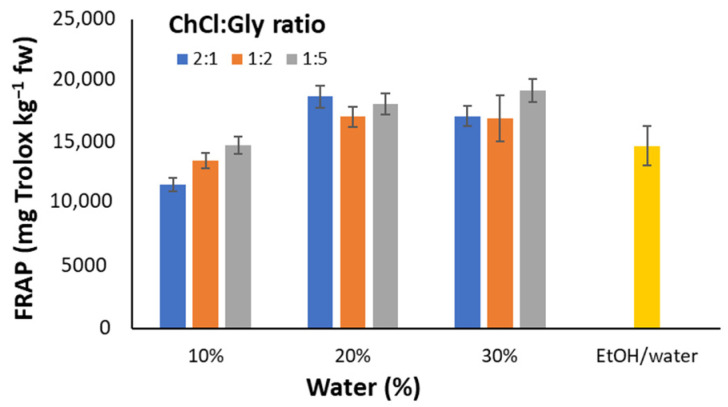
Influence of ChCl:Gly ratio (2:1, 1:2, and 1:5) and water percentage (10%, 20%, and 30%) on the overall antioxidant capacity by FRAP. Results are compared with that from EtOH/water (20:80, *v*/*v*) extract as the reference of conventional solvent. Error bars indicate the standard deviations.

The chromatographic profiles obtained were considered to evaluate the effect of the ChCl:Gly ratio and the percentage of water on the extraction efficiency. For simplicity, the total area (between 10–25 min) as well as peak areas of three of the most prominent peaks, identified as Luteolin-7-O-glucoside, Apigenin-7-O-rutinoside, and Luteolin-7-O-glucoside isomer, were compared at the different conditions assayed. In general, the concentration of polyphenolic compounds slightly increases as the percentage of water increases for the same molar ratio. Similar trends are observed with the antioxidant capacity measured with the FRAP index (Figure 2). FRAP results show that the percentage of water influences the extraction yield while the HBA/HBD ratio does not affect the reducing power. Regarding the DPPH assay, no significant differences are observed at all. As a result, and based on extraction performance, solvent cost, and easiness of preparation, a NADES composition of ChCl:Gly 1:5 (*m:m*) and 30% water was selected for further studies.

**Figure 3:**
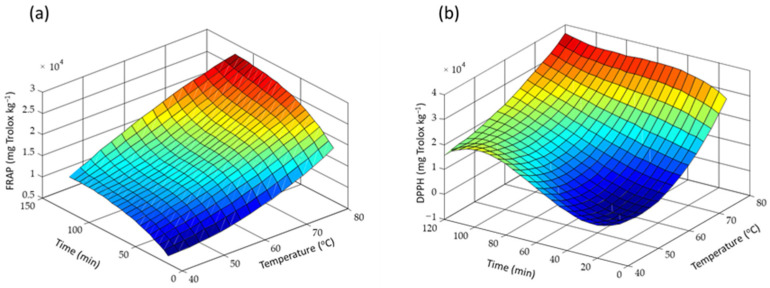
Effect of temperature and extraction time on (**a**) the antioxidant capacity (FRAP) and (**b**) the radical scavenger capacity (DPPH).

Once the solvent composition was established, the influence of temperature and process time on the extraction was evaluated under the selected NADES composition—ChCl:Gly 1:5 (*m:m*) and 30% water—and constant magnetic stirring. The variables were optimized with an experimental grid design at three temperatures and four times, thus resulting in twelve runs. The evaluated temperatures were 40 °C, 60 °C, and 80 °C, and the extraction times were 15 min, 30 min, 60 min, and 120 min. Each condition was assayed in duplicate. As previously, the performance of the extraction was evaluated by comparing the antioxidant indexes and the chromatographic profiles at the different studied conditions. As can be seen in Figure 3, the antioxidant capacity, both FRAP and DPPH indexes, increases with increasing the temperature and the extraction time, obtaining the maximum antioxidant capacity at 80 °C and 120 min. In the same line, the total peak area (between 10 and 25 min) and peak area for Luteolin-7-O-glucoside, Apigenin-rutinoside, and Luteolin-7-O-glucoside isomer increased when the temperature increased, obtaining the highest outcomes (around a 50% increment) from 60 °C to 80 °C. These data also show that at 80 °C, there is no significant temperature degradation of the studied polyphenolic compounds.

From these graphs, it was deduced that higher processing times and temperatures could improve extraction performance. However, previous studies dealing with other olive oil waste matrices suggested that some polyphenols begin to degrade when subjected to higher temperatures or extraction times [32]. In addition, from a practical point of view, considering further applications at the pilot plant or industrial level, an increase in time or temperature has a negative impact on production costs, so here it is considered that the selected conditions result in a good compromise solution between extraction performance, process time, and energy costs.

In summary, the extraction of polyphenols from olive tree leaves was optimized using NADES as a green alternative to conventional solvents. The optimum conditions were Ch:Gly 1:5 with 30% water, 80 °C extraction temperature, and 2 h process time with constant magnetic stirring.

**Figure 4:**
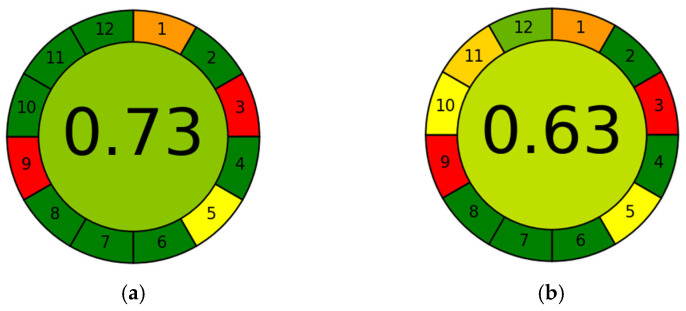
Graphs obtained with AGREE: (**a**) extraction with NADES and (**b**) extraction with ethanol/water. Identification of numbers: 1, Avoid Sample Treatment; 2, Minimal Sample Size; 3, In Situ Measurements; 4, Save Reagents; 5, Automated and Miniaturized Methods; 6, Derivatization Avoided; 7, Waste Avoided; 8, Multianalyte Methods; 9, Energy Minimized; 10, Reagents from Renewable Source; 11, Toxic Reagents Eliminated; 12, Operator Safety.

In order to compare the two extraction methods (i.e., the one using hydro-organic mixtures and the one using the optimum NADES) from a sustainability and environmental point of view, both methods were analyzed with the AGREE (Analytical GREennEss calculator) software [33]. This software allows us to evaluate, from 0 to 1, the greenness of analytical procedures considering 12 principles of green analytical chemistry. As can be seen in Figure 4, the extraction with NADES achieves a better overall score in greenness, showing that this method is greener and safer for human health and the ecosystem than the one using conventional hydro-organic solvents.

### 3.3. Determination of Phenolic Compounds in the Olive Leaf ChCl:Gly Extract

**Figure 5:**
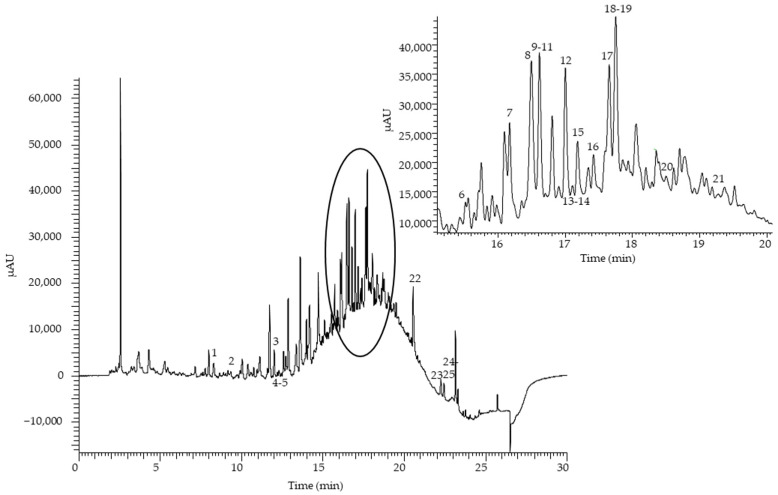
HPLC-UV chromatogram of an olive tree leaf NADES extract recorded at 280 nm. For peak identification, see Table 1.

This study only intended to have a general idea of the most abundant compounds and the approximate concentration levels for this type of matrix. An exhaustive identification study has been carried out, but levels in different similar residues have not been quantified depending on olive varieties, processing, etc. The study only intends to show what type of NADES and composition is more efficient for the extraction of analytes and if the procedure based on NADES improves the performance of conventional solvents. Hence, the olive leaf extract obtained by mechanical stirring at 80 °C for 120 min using ChCl:Gly (1:5 ratio with 30% water) as the solvent had an antioxidant capacity of 27,700 and 36,500 mg eq. Trolox kg^−1^ fw estimated by the FRAP and the DPPH methods, respectively. The relative standard deviation (RSD%) of three independent replicates (*n* = 3) was 6.3% and 3.4% for FRAP and DPPH, respectively. Regarding the chromatographic profile, the LC-UV chromatogram at 280 nm is complex, with a large number of peaks, most of them attributable to phenolic compounds (Figure 5).

For quantification purposes, the final extract was analyzed by LC-MS/MS working in MRM mode (Table 3). Quantitative results show that 3-Hydroxytyrosol, Oleuropein, and Luteolin-7-O-glucoside are the most important polyphenols in olive tree leaves. These compounds present concentrations between 130 and 260 mg kg^−1^ of fresh olive tree leaves. The variability in the determination of these major analytes established by independent extractions is satisfactory, with RSD% values below 5%. Similar results were reported by other authors using conventional solvents and NADES as extractants [20,34,35,36]. Other relevant components are Luteolin and Rutin with concentrations around 30 mg kg^−1^ of fresh olive tree leaves and Verbascoside and 3,4-Dihydroxybenzoic acid at 15 mg kg^−1^ of fresh olive tree leaves. The rest of the quantified polyphenols compounds were found in concentrations lower than 10 mg kg^−1^ of fresh olive tree leaves (with RSD values in general below 15%). Among them, Caffeic acid, Apigenin, or Ferulic acid are also extracted at a similar extent using conventional solvents [37].

As mentioned, the olive oil production industry generates thousands of tons of waste that, from the point of view of the circular economy, can go from being a problem to an opportunity. In this sense, the use of NADES for the extraction of bioactive compounds from this type of matrices, particularly the extraction of phenolic compounds from olive leaf residues, is viewed as an excellent approach for their revalorization as a source of notable compounds, such as derivatives of Luteolin and Oleuropein. From the point of view of bioactive compounds, all these compounds, especially the most abundant ones, belong to the families of phenylethanoids (hydroxytyrosol and derivatives), hydroxycinnamic acids, and flavonoids (especially flavones). These compounds stand out for their remarkable antioxidant capacity, providing complementary cardioprotective anticancer, anti-inflammatory, neuroprotective, and antimicrobial properties [38,39]. From a quantitative point of view, Luteolin-7-O-glucoside, Oleuropein, and 3-Hydroxytyrosol are by far the most remarkable molecules, with more than 100 mg kg^−1^ fw. All of them have abundant information in the literature in which their properties are discussed. This information has been compiled in several excellent reviews [40,41]. Apart from their undoubted potential in the pharmaceutical and food industry, their role as building blocks in the chemical industry and their applications as innovative functional foods also deserve to be highlighted [42,43].

## 4. Conclusions

The characterization of the phenolic profile in olive tree leaves by liquid chromatography coupled with mass spectrometry identified the most relevant phenolic compounds occurring in this agri-food waste. Various NADES combinations were prepared using choline chloride as the hydrogen bond acceptor and glycerol, urea, and lactic acid as the donors and were assessed in terms of the extraction efficiency of phenolic compounds. Despite the fact that the extraction performance of the NADES systems seemed to depend on each compound of interest, overall, the choline chloride/glycerol system was more efficient for most target analytes. Urea was also an efficient donor, which provided excellent overall polyphenolic extractions, especially great antioxidant capacities. The optimization of the extraction conditions relied on experimental design, considering choline/glycine molar ratio, water percentage, temperature, and process time as potential factors. The optimum conditions were Ch:Gly 1:5 with 30% water, 80 °C extraction temperature, and a 2 h process time with magnetic stirring. NADES resulted in a good alternative to conventional solvents from an environmental and sustainability point of view, also providing a more efficient extraction. For this reason, further research is needed in this area concerning the purification of the resulting extracts to generate by-products suitable for different applications in cosmetics, nutraceuticals, or even in packaging.

## Figures and Tables

**Table 1:** Phenolic compounds identified in olive tree leaves extracts.

Peak No.	Retention Time (min)	Measured [M − H]^−^ *m*/*z*	Tentative Formula	Mass Error (ppm)	Identification
1	8.50	153.0557	C_8_H_10_O_3_	−0.1	3-Hydroxytyrosol
2	9.19	153.0194	C_7_H_6_O_4_	0.5	3,4-Dihydroxibenzoic acid
3	11.98	389.1105	C_16_H_22_O_11_	3.9	Secologanoside ^a^
4	12.44	137.0247	C_7_H_6_O_3_	1.8	4-Hydroxybenzoic acid
5	12.47	353.0887	C_16_H_18_O_9_	2.4	Chlorogenic acid
6	15.54	151.0403	C_8_H_8_O_3_	1.6	Vanillin
7	16.23	609.1480	C_27_H_30_O_16_	3.0	Rutin
8	16.48	163.0406	C_9_H_8_O_3_	3.2	p-Coumaric acid
9	16.69	447.0953	C_21_H_20_O_11_	4.6	Luteolin-7-O-glucoside
10	16.69	701.2317	C_31_H_42_O_18_	2.6	Oleuropein glucoside ^a^
11	16.72	463.0897	C_21_H_20_O_12_	3.2	Quercetin-3-O-glucoside
12	17.07	577.1585	C_27_H_30_O_14_	3.8	Apigenin-7-O-rutinoside ^a^
13	17.10	193.0600	C_10_H_10_O_4_	1.6	Ferulic acid
14	17.14	607.1685	C_28_H_32_O_15_	2.7	Diosmin
15	17.24	609.1823	C_28_H_34_O_15_	−0.3	Hesperidin
16	17.49	623.2001	C_29_H_36_O_15_	3.2	Verbascoside
17	17.56	539.1790	C_25_H_32_O_13_	3.7	Oleuropein
18	17.68	447.0944	C_21_H_20_O_11_	2.5	Luteolin-7-O-glucoside isomer ^a^
19	17.70	431.0999	C_21_H_20_O_10_	3.5	Apigenin-7-O-glucoside ^a^
20	18.50	377.1245	C_19_H_22_O_8_	0.8	Oleuropein aglycone
21	19.27	523.1840	C_25_H_32_O_12_	3.6	Ligstroside ^a^
22	20.64	285.0415	C_15_H_10_O_6_	3.7	Luteolin
23	22.24	271.0617	C_15_H_12_O_5_	2.0	Naringenin
24	22.32	269.0470	C_15_H_10_O_5_	3.8	Apigenin

^a^: tentative assignation, standard not available.

**Table 2:** Relative composition of the NADES and EtOH/water extracts (ChCl:Gly is used as the reference).

	ChCl:Gly (1:2, *m:m*; 10% water)	ChCl:Urea (1:2, *m:m*, 10% water)	ChCl:Lactic (1:2, *m:m*; 10% water)	EtOH/water (20:80, *v*/*v*)
3,4-Dihydroxybenzoic acid	1.0	1.7	0.1	<0.1
3-Hydroxytyrosol	1.0	0.3	1.1	0.3
Apigenin	1.0	0.4	0.6	0.2
Astilbin	1.0	1.7	0.7	<0.1
Caffeic acid	1.0	<0.1	0.4	0.2
Chlorogenic acid	1.0	1.4	0.5	0.3
Diosmin	1.0	1.8	0.9	0.5
Ferulic acid	1.0	0.5	0.4	0.7
Hesperidin	1.0	<0.1	0.8	<0.1
Luteolin	1.0	0.1	0.4	<0.1
Luteolin-7-O-glucoside	1.0	1.7	0.7	0.6
Naringenin	1.0	1.0	0.5	<0.1
Naringin	1.0	2.1	0.6	0.4
Oleuropein	1.0	0.4	<0.1	0.9
Oleuropein aglycone	1.0	<0.1	0.2	0.3
p-Coumaric acid	1.0	1.0	0.5	0.4
Quercetin	1.0	<0.1	1.4	<0.1
Quercetin-3-O-glucoside	1.0	1.4	0.4	0.3
Rutin	1.0	1.7	0.5	0.7
Vanillin	1.0	0.8	0.4	<0.1
Verbascoside	1.0	<0.1	1.3	<0.1

**Table 3:** Concentration (mg kg^−1^ fresh olive tree leaves) of the main phenolic compounds identified in olive leaf NADES extracts.

Phenolic Compound	Concentration (mg kg fw^−1^)
Luteolin-7-O-glucoside	262.0
Oleuropein	173.3
3-Hydroxytyrosol	128.6
Rutin	32.7
Luteolin	28.7
3,4-dihydroxybenzoic acid	14.8
Verbascoside	14.2
Diosmin	7.9
Naringin	6.9
Quercetin-3-O-glucoside	5.9
p-Coumaric acid	5.3
Apigenin	2.7
Ferulic acid	2.2
Chlorogenic acid	2.0
Caffeic acid	1.2
Hesperidin	1.0
Naringenin	0.6

## Data Availability

Not applicable.

## Supplementary Materials

The following supporting information can be downloaded at: https://www.mdpi.com/article/10.3390/antiox12050995/s1, Figure S1: HRMS (a) and HRMS/MS (b) spectra of Oleuropein standard and HRMS (c) and HRMS/MS (d) spectra of Oleuropein in the olive tree leaves extracts (chromatographic peak at 17.6 min). (*) The peak corresponding to the [M − H]^−^ ion of Luteolin-7-O-glucoside isomer, which coelutes with Oleuropein; Table S1: MRM transitions for the detection of polyphenols by LC-ESI-MS/MS.
