# Supplementary material for: Green Extraction of Antioxidant Compounds from Olive Tree Leaves Based on Natural Deep Eutectic Solvents
Source: Antioxidants (Basel). 2023 Apr 25;12(5):995. doi: 10.3390/antiox12050995 (PMC10215426; doi:10.3390/antiox12050995)
Supplement: Supplementary file 1 [file antioxidants-12-00995-s001.zip › antioxidants-2328790-supplementary.pdf]

Green Extraction of Antioxidant Compounds from Olive Tree Leaves Based on Natural Deep Eutectic Solvents

Aina Mir-Cerdà [1,2], Mercè Granados [1], Javier Saurina [1,2] and Sonia Sentellas [1,2,3,*]

[1] Department of Chemical Engineering and Analytical Chemistry, Universitat de Barcelona, Martí i Franquès 1-11, E08028 Barcelona, Spain
[2] Research Institute in Food Nutrition and Food Safety, Universitat de Barcelona, Av. Prat de la Riba 171, Edifici Recerca (Gaudí), E08921 Santa Coloma de Gramenet, Spain
[3] Serra Húnter Fellow Programme, Generalitat de Catalunya, Via Laietana 2, E08003 Barcelona, Spain
* Correspondence: sonia.sentellas@ub.edu; Tel.: +34-93-403-54-23

Table S1. MRM transitions for the detection of polyphenols by LC-ESI-MS/MS.

Phenolic compound	Parent ion (*m/z*)	Daughter ion (*m/z*)	DP (volts)	EP (volts)	CE (volts)	CXP (volts)
**(-)-Epigallocatechin**	305.0	136.9	-80	-6.5	-36	-10
**2,5-Dyhydroxybenzoic acid**	152.9	108.9	-70	-10	-20	-3
**3,4-Dyhydroxybenzoic acid**	152.9	108.9	-70	-10	-20	-3
**3-Hydroxytyrosol**	152.9	122.9	-35	-7	-22	-8
**4-Hydroxybenzoic acid**	136.8	92.9	-55	-10	-18	-7
**Apigenin**	268.9	116.9	-80	-10	-52	-1
**Astilbin**	449.1	285.0	-100	-10	-32	-1
**Caffeic Acid**	178.8	133.8	-60	-10	-33	-7
**Caftaric acid**	311.1	179.0	-50	-10	-22	-11
**Catechin**	289.0	108.8	-65	-10	-34	-15
**Chlorogenic acid**	352.9	190.6	-60	-10	-20	-17
**Chrysin**	253.0	62.9	-75	-10	-58	-1
**Diosmin**	607.4	299.1	-105	-10	-34	-23
**Ellagic acid**	300.9	173.1	-108	-10	-50	-5
**Epicatechin**	289.0	108.8	-65	-10	-34	-15
**Ethyl gallate**	197.2	123.8	-60	-10	-34	-3
**Ferulic Acid**	192.8	134.0	-43	-11	-21	-8
**Fisetin**	285.0	134.8	-70	-10	-30	-19
**Galangin**	268.9	41.1	-80	-10	-80	-3
**Gallic acid**	169.0	124.9	-40	-10	-22	-19
**Hesperidin**	609.3	301.2	-115	-10	-36	-19
**Hesperetin**	301.0	163.8	-85	-10	-36	-1
**Quercetin-3-O-glucoside**	463.1	300.9	-90	-10	-38	-37
**Kaempferol**	285.1	117.0	-91	-10	-63	-3
**Ligstroside**	523.0	291.0	-103	-5	-33	-10
**Luteolin**	284.9	132.8	-85	-10	-48	-17
**Luteolin-7-glucoside**	447.3	283.9	-108	-10	-52	-17
**Myricetin**	316.9	150.8	-75	-10	-38	-21
**Naringenin**	271.0	150.8	-25	-10	-28	-7
**Naringin**	579.3	271.0	-52	-10	-52	-7
**Oleacein**	318.8	194.8	-85	-8	-12	-9
**Oleocanthal**	303.0	179.0	-70	-5	-11	-6
**Oleuropein**	539.2	275.0	-35	-10	-32	-9
**Oleuropein Aglycone**	376.9	274.9	-40	-10	-18	-11
**p-Coumaric Acid**	163.0	119.0	-50	-5	-19	-8
**Pinocembrin**	255.1	150.9	-75	-10	-34	-21
**Procyanidin A2**	575.2	285.2	-135	-10	-38	-1
**Procyanidin B1**	577.3	288.9	-90	-10	-40	-15
**Procyanidin B2**	577.1	407.1	-105	-10	-26	-11
**Procyanidin C2**	865.3	125.1	-180	-10	-86	-7
**Procyanidin C1**	865.3	407.0	-110	-10	-70	-17
**Quercetin**	300.9	150.8	-80	-10	-32	-1
**Resveratrol**	227.0	184.8	-60	-10	-28	-7
**Rutin**	609.2	301.2	-115	-10	-52	-43

**Synapic Acid**	223.0	163.8	-45	-10	-22	-9
**Syringic Acid**	196.9	181.9	-20	-11	-19	-6
**trans-Cinnamic acid**	147.2	102.6	-50	-10	-16	-17
**Trans-Coutaric acid**	295.0	162.9	-30	-10	-20	-11
**Tyrosol**	136.8	107.0	-82	-11	-23	-4
**Vanillic acid**	166.9	151.8	-65	-10	-20	-1
**Vanillin**	150.9	135.9	-50	-10	-14	-31
**Verbascoside**	623.2	160.9	-95	-10	-58	-23

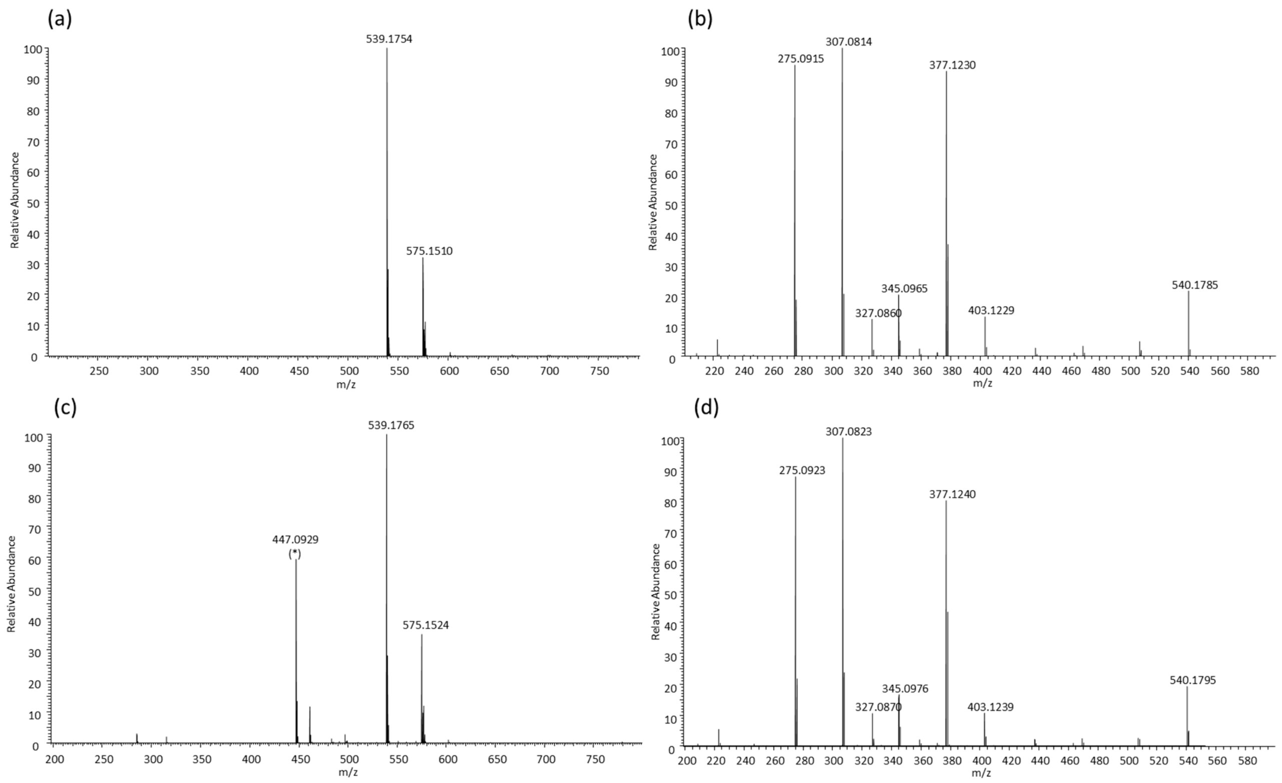

Figure S1. HRMS (a) and HRMS/MS (b) spectra of oleuropein standard and HRMS (c) and HRMS/MS (d) spectra of oleuropein in the olive tree leaves extracts (chromatographic peak at 17.6 min). (*) peak corresponding to the $[M-H]^-$ ion of luteolin-7-O-glucoside isomer which coelute with oleuropein.
